# Supplementary material for: Mendelian Randomization Analysis Reveals Causal Effects of Polyunsaturated Fatty Acids on Subtypes of Diabetic Retinopathy Risk
Source: Nutrients. 2023 Sep 29;15(19):4208. doi: 10.3390/nu15194208 (PMC10574403; doi:10.3390/nu15194208)
Supplement: Supplementary file 1 [file nutrients-15-04208-s001.zip › STROBE-MR-checklist-fillable.pdf]

STROBE-MR checklist of recommended items to address in reports of Mendelian randomization studies[1 2]

Item No.	Section	Checklist item	Page No.	Relevant text from manuscript
1	**TITLE and ABSTRACT**	Indicate Mendelian randomization (MR) as the study's design in the title and/or the abstract if that is a main purpose of the study	1	Causal associations between polyunsaturated fatty acids and diabetic retinopathy: A two-sample Mendelian randomization study
	**INTRODUCTION**			
2	**Background**	Explain the scientific background and rationale for the reported study. What is the exposure? Is a potential causal relationship between exposure and outcome plausible? Justify why MR is a helpful method to address the study question	3, 4	Diabetic retinopathy (DR)，which typically includes non-proliferative diabetic retinopathy (NPDR) and proliferative diabetic retinopathy (PDR), is the main ocular consequence of diabetes mellitus and affects between 30 and 40 percent of diabetics. The sudden rise in the prevalence of DR diseases by more than 25% in just 10 years is likely to put additional strain on already overburdened healthcare systems and resources. The financial expenses of DR and its complications are substantial.<br>Polyunsaturated fatty acids (PUFAs), which comprise omega-3 fatty acids (FAw3) and omega-6 fatty acids (FAw6), have been reported to impact physiological processes, including anti-inflammatory, immunological, glucose and lipid metabolism, and visual acuity. More recently, higher PUFAs, particularly FAw3, have been found to be associated with a reduced risk of DR progression, while controversies persist regarding the effects of FAw6.<br>However, serum lipid profiles are significantly altered by variables like fasting and medication use independent from the underlying disease, and traditional conventional studies could not completely rule out confounding bias or reverse causality. Randomized controlled trials (RCTS) are typically regarded as the gold standard in clinical evidence15, but to date, no RCTs have been reported to study the effect of PUFAs on DR, possibly due to the difficulties in meeting the criteria of sample size and intervention duration.<br>Mendelian randomization (MR) has become a popular and practical approach employed mainly in

				epidemiological etiological inference in recent years, and can imitate RCTS without much difficulty. It employs genetic variation as an instrumental variable (IVs) to assess causality between biomarkers and outcomes. Thanks to taking advantage of the natural randomization of genetic variation, it avoids the problems of confounding factors and reverse causation, and can be considered to serve as an appropriate approach to evaluate the relationship between serum lipid and diseases of concern.
3	**Objectives**	State specific objectives clearly, including pre-specified causal hypotheses (if any). State that MR is a method that, under specific assumptions, intends to estimate causal effects	5	In this study, we performed a bidirectional causal effects between totally PUFAs and ADR and a 2-sample MR study to comprehensively investigate the potential possible causality of different types of PUFAs (totally PUFAs, FAw3, FAw6, and FAw6/FAw3), on three DR phenotypes, 1) background diabetic retinopathy (BDR), 2) severe non-proliferative diabetic retinopathy (SNPDR), and 3)PDR, to assess the associations between PUFAs and DR.
	**METHODS**			
4	**Study design and data sources**	Present key elements of the study design early in the article. Consider including a table listing sources of data for all phases of the study. For each data source contributing to the analysis, describe the following:		
	a)	Setting: Describe the study design and the underlying population, if possible. Describe the setting, locations, and relevant dates, including periods of recruitment, exposure, follow-up, and data collection, when available.	5	We performed a bidirectional MR analysis based on the summary genetic associations from different Genome-Wide Association Study (GWAS) for totally PUFAs and ADR. Then, we used 2-sample MR to examine the the associations of different genetically determined pufatraits of PUFAs (totally PUFA, FAw3, FAw6, ratio of FAw6 to FAw3) with four phenotypes of DR (BDR, SNPDR, PDR), respectively.<br><br>According to the three core assumptions of MR: (i) the "correlation" hypothesis, where IV is closely related to exposure; (ii) the assumption of "independence", where IV is not related to confounders; (iii) the "exclusion limitation" hypothesis, where IV does not affect results by means other than exposure, namely no horizontal pleiotropy19, SNPs representing IVs after

			screening were selected. A brief description of the MR design is displayed in Figure 1.
b)	Participants: Give the eligibility criteria, and the sources and methods of selection of participants. Report the sample size, and whether any power or sample size calculations were carried out prior to the main analysis	6, 7	PUFAs' GWAS contained 114999 samples, all of which were from Europe (36 samples) and ranged in age from 40-69 years20. This GWAS explains 4.8-7.9% of the variance of the cyclic PUFAs. In this study, 4 pufatraits were selected as exposure amounts: total PUFAs, circulating FAw3, FAw6, ratio of FAw6 to FAw3. The mean concentrations of circulating fatty acids of the UK Biobanks participants were 0.53 mmol/L (SD 0.22) and 4.45 mmol/L(SD 0.68) of total FAw3 and total FAw6, respectively, accordingly accounting for 4.4% and 38% of total fatty acids.<br><br>The DR GWAS data can be downloaded from the FinnGen research project (https://r5.finngen.fi/)21. DR was identified by using the ICD-10 code (H36.0 or E11.3). 16962023 variables were analyzed from 218792 subjects through the gWAS SAIGE (https://github.com/weizhouumich/SAIGE). According to different severity of DR, four DR phenotypes were slected as exposure amounts: ADR (ICD 10: H36.0*; 18,097 cases, 206,364 controls), BDR (ICD 10: H36.00; 2510 cases, 242,308 controls), SNPDR (ICD 10: H36.02; 568 cases, 242,308 controls), and PDR (ICD 10: H36.03; 10,860 cases, 242,308 controls).<br><br>After adjusting for a group of possible confounder of age, sex, genotyping batches, genetic correlation, diabetes course, hypertension and cardiovascular history, and average daily dosage of hypoglycemic agents after cohort entry. More details in FinnGen were described in https://finngen.gitbook.io/documentation/v/r7/.<br><br>The FinnGen Study is a Finnish national meta-analysis of GWAS in 9 biobanks and has much limited overlap with UK biobanks GWAS in several centres in the UK. Therefore, we believe that sample overlap between PUFAs and DR Data leads to minimal risk of bias.
c)	Describe measurement, quality control and selection of genetic variants	7	To satisfy three core assumptions of MR, all SNPs were screened from the GWAS data ($P<5\times10^{-8}$) at the genome-wide significance level. Secondly, using the “ld_clump” R package, linkage disequilibrium between SNPs ($R^2<0.001$, and

					<10000 from the index variant) was identified. We aligned effect alleles of outcome-related SNPs with effect alleles of exposure-related SNPS based on allelic letters and allelic frequencies, and removed the SNP alleles with a palindrome. Finally, we used PhenoScanner database (https://www.phenoscanner.medschl.cam.ac.uk/) to verify whether the SNP loci is associated with other confounding factors.
		d)	For each exposure, outcome, and other relevant variables, describe methods of assessment and diagnostic criteria for diseases	6	After adjusting for a group of possible confounder of age, sex, genotyping batches, genetic correlation, diabetes course, hypertension and cardiovascular history, and average daily dosage of hypoglycemic agents after cohort entry. More details in FinnGen were described in https://finngen.gitbook.io/documentation/v/r7/.
		e)	Provide details of ethics committee approval and participant informed consent, if relevant	17	Ethical approval was not sought for this specific project because all data came from the summary statistics of published GWAS, and no individual-level data were used.
5	**Assumptions**		Explicitly state the three core IV assumptions for the main analysis (relevance, independence and exclusion restriction) as well assumptions for any additional or sensitivity analysis	5, 8, 9	According to the three core assumptions of MR: (i) the "correlation" hypothesis, where IV is closely related to exposure; (ii) the assumption of "independence", where IV is not related to confounders; (iii) the "exclusion limitation" hypothesis, where IV does not affect results by means other than exposure, namely no horizontal pleiotropy19, SNPs representing IVs after screening were selected.<br><br>We corrected horizontal pleiotropy by detecting and removing outliers through REsidual Sum and Outlier (MR-PRESSO) test, and determined whether there was a substantial change in causality before and after removing outliers. Then MR-Egger regression was used to evaluate the possibility of average horizontal pleiotropy of IVs, and funnel charts to visualize directional pleiotropy. If the MR-egger intercept has no statistical significance (P>0.05) and relative symmetry of funnel plots can also be considered as an indicator of horizontal pleiotropy.<br><br>Furthermore, Cochran's Q test (P<0.05 indicates heterogeneity) was used to assess heterogeneity between SNPs in IVW estimates. In older to detect

					the robustness and consistency of the results, we utilized a “leave-one-out” sensitivity analysis to where the MR is performed again but leaving out each SNP in turn.
6	**Statistical methods: main analysis**		Describe statistical methods and statistics used		
		a)	Describe how quantitative variables were handled in the analyses (i.e., scale, units, model)	7, 8	In our study, we applied three complementary approaches, the inverse variance weighted (IVW), the MR-Egger regression, and the Weighted Median (WM), to estimate the causal effects of exposures on outcomes.<br>IVW was used as the primary outcome, which can take the inverse variance of each study as the weight, calculate the weighted average of the effect size, and summarize the effect size of multiple independent studies. When all the selected SNPs are valid IVs, the most accurate estimation results can be provided26. Since WM and MR-Egger could provide more reliable but albeit less efficient estimates over a wider set of scenarios, they were used to improve IVW estimates27, 28. Finally, Causal estimates were expressed as odds ratios (ORs) with 95% confidence intervals (CIs).
		b)	Describe how genetic variants were handled in the analyses and, if applicable, how their weights were selected	7	F statistics for these IVs were calculated before MR analysis to determine if there was a weak IV bias. For all IVs, F>10, respectively, the effect of weak IV bias is little, so the selected SNPs can be further used in MR study.
		c)	Describe the MR estimator (e.g. two-stage least squares, Wald ratio) and related statistics. Detail the included covariates and, in case of two-sample MR, whether the same covariate set was used for adjustment in the two samples	7	The FinnGen Study is a Finnish national meta-analysis of GWAS in 9 biobanks and has much limited overlap with UK biobanks GWAS in several centres in the UK. Therefore, we believe that sample overlap between PUFAs and DR Data leads to minimal risk of bias.
		d)	Explain how missing data were addressed	7	We aligned effect alleles of outcome-related SNPs with effect alleles of exposure-related SNPs based on allelic letters and allelic frequencies, and removed the SNP alleles with a palindrome. Finally, we used PhenoScanner database (https://www.phenoscanner.medschl.cam.ac.uk/) to

					verify whether the SNP loci is associated with other confounding factors.
		e)	If applicable, indicate how multiple testing was addressed		
7	**Assessment of assumptions**		Describe any methods or prior knowledge used to assess the assumptions or justify their validity	7	F statistics for these IVs were calculated before MR analysis to determine if there was a weak IV bias. For all IVs, F>10, respectively, the effect of weak IV bias is little, so the selected SNPs can be further used in MR study.
8	**Sensitivity analyses and additional analyses**		Describe any sensitivity analyses or additional analyses performed (e.g. comparison of effect estimates from different approaches, independent replication, bias analytic techniques, validation of instruments, simulations)	8	Cochran's Q test (P<0.05 indicates heterogeneity) was used to assess heterogeneity between SNPs in IVW estimates. In older to detect the robustness and consistency of the results, we utilized a "leave-one-out" sensitivity analysis to where the MR is performed again but leaving out each SNP in turn31.
9	**Software and pre-registration**				
		a)	Name statistical software and package(s), including version and settings used	8	"TwoSampleMR" was performed in all analysis software package (https://github.com/mrcieu/TwoSampleMR) and "MR-presSO" package (statistical computing internal resistance project) 4.2.0 version in R (version 3.6.1) packages.
		b)	State whether the study protocol and details were pre-registered (as well as when and where)		
	**RESULTS**				
10	**Descriptive data**				
		a)	Report the numbers of individuals at each stage of included studies and reasons for exclusion. Consider use of a flow diagram	10	Following a rigorous screening process based on the independence and exclusivity hypotheses, as well as the harmonization and removal of palindromic SNPs with intermediate allele frequencies, we meticulously selected 52 SNPs for the MR analysis to assess the associations between total PUFA and ADR. Similarly, for the evaluation of the relationship between ADR and total PUFA, 8 SNPs were selected as instrumental variables. The study also focused on investigating the causal effects of different types of PUFAs, including total PUFA, FAw3, FAw6, and

				FAw6/FAw3, on three DR phenotypes (BDR, SNPDR, and PDR) using MR analysis. In total, 34-57 SNPs were chosen for the MR analysis. The F-statistics of the instruments exceeded 10 for all selected SNPs, indicating satisfactory instrument strength. Detailed information regarding the instruments can be found in Supplemental Table 1.
		b) Report summary statistics for phenotypic exposure(s), outcome(s), and other relevant variables (e.g. means, SDs, proportions)	10	Following a rigorous screening process based on the independence and exclusivity hypotheses, as well as the harmonization and removal of palindromic SNPs with intermediate allele frequencies, we meticulously selected 52 SNPs for the MR analysis to assess the associations between total PUFA and ADR. Similarly, for the evaluation of the relationship between ADR and total PUFA, 8 SNPs were selected as instrumental variables. The study also focused on investigating the causal effects of different types of PUFAs, including total PUFA, FAw3, FAw6, and FAw6/FAw3, on three DR phenotypes (BDR, SNPDR, and PDR) using MR analysis. In total, 34-57 SNPs were chosen for the MR analysis. The F-statistics of the instruments exceeded 10 for all selected SNPs, indicating satisfactory instrument strength. Detailed information regarding the instruments can be found in Supplemental Table 1.
		c) If the data sources include meta-analyses of previous studies, provide the assessments of heterogeneity across these studies		
		d) For two-sample MR:<br>i. Provide justification of the similarity of the genetic variant-exposure associations between the exposure and outcome samples<br>ii. Provide information on the number of individuals who overlap between the exposure and outcome studies	7	The FinnGen Study is a Finnish national meta-analysis of GWAS in 9 biobanks and has much limited overlap with UK biobanks GWAS in several centres in the UK. Therefore, we believe that sample overlap between PUFAs and DR Data leads to minimal risk of bias.
11	**Main results**			
		a) Report the associations between genetic variant and exposure, and between genetic variant and outcome, preferably on an interpretable scale	10	The results of MR showed that genetically predicted totally PUFA was associated with an decreased risk of ADR. The MR analysis results showed that genetically predicted totally PUFAs was associated with a decreased risk of BDR and PDR, while FAw3 was associated with a decreased risk of PDR, and FAw6 was associated with a decreased risk of BDR and PDR

		b) Report MR estimates of the relationship between exposure and outcome, and the measures of uncertainty from the MR analysis, on an interpretable scale, such as odds ratio or relative risk per SD difference	10, 11	The results of MR showed that genetically predicted totally PUFA was associated with an decreased risk of ADR (IVW: OR, 0.90; 95% CI, 0.83-0.97; P=5.05×10-3). It can be seen from the scatter plot (Figure 2) that the causal effect among the three methods is consistent. However, no association of ADR with totally PUFAs was found using any method (all P>0.05). The MR analysis results showed that genetically predicted totally PUFAs was associated with a decreased risk of BDR (IVW: OR, 0.79; 95% CI, 0.65-0.98; P=2.90×10-2) and PDR (IVW: OR, 0.86; 95% CI, 0.78-0.95; P=2.63×10-3), while FAw3 was associated with a decreased risk of PDR (IVW: OR, 0.83; 95% CI, 0.74-0.93; P=1.50×10-3), and FAw6 was associated with a decreased risk of BDR (IVW: OR, 0.69; 95% CI, 0.56-0.86; P=9.20×10-4) and PDR (IVW: OR, 0.82; 95% CI, 0.74-0.91; P=3.51×10-4). The causal effects among the three methods were consistent, as shown in the scatter plot (Figure 2). However, no association of any of the three fatty acids with SNPDR was found using any method (all P>0.05). In addition, no association of the combined FAw6/FAw3 with any of the DR phenotypes was found using any method (all P>0.05). Further details on the MR analysis are provided in Figure 3.
		c) If relevant, consider translating estimates of relative risk into absolute risk for a meaningful time period		
		d) Consider plots to visualize results (e.g. forest plot, scatterplot of associations between genetic variants and outcome versus between genetic variants and exposure)	10, 11	It can be seen from the scatter plot (Figure 2) that the causal effect among the three methods is consistent. More MR analysis details are shown in Figure 3.
12	**Assessment of assumptions**			
		a) Report the assessment of the validity of the assumptions	7, 8	In our study, we applied three complementary approaches, the inverse variance weighted (IVW), the MR-Egger regression, and the Weighted Median (WM), to estimate the causal effects of exposures on outcomes.<br>IVW was used as the primary outcome, which can take the inverse variance of each study as the weight, calculate the weighted average of the effect

					size, and summarize the effect size of multiple independent studies. When all the selected SNPs are valid IVs, the most accurate estimation results can be provided26. Since WM and MR-Egger could provide more reliable but albeit less efficient estimates over a wider set of scenarios, they were used to improve IVW estimates27, 28. Finally, Causal estimates were expressed as odds ratios (ORs) with 95% confidence intervals (CIs).<br>"TwoSampleMR" was performed in all analysis software package (https://github.com/mrcieu/TwoSampleMR) and "MR-presSO" package (statistical computing internal resistance project) 4.2.0 version in R (version 3.6.1) packages. For the Bidirectional MR of totally PUFAs and ADR, a global-level test, a significant two-sided P-value was set as 0.05. The Bonferroni correction, a conservative method, was used to examine the associations between 3 exposures (i.e., totally PUFAs, FAw3, FAw6, and FAw6/FAw3) and 3 DR outcomes (i.e., BDR, SNPDR, and PDR). Therefore, for region-level analyses, a Bonferroni-corrected P-value<4.17×10-3 [0.05/(3*4)] on both sides is regarded as significant, and P-value<0.05 on both sides is regarded as nominally significant. For a global-level test, a nominally significant two-sided P-value was set as 0.05.
		b)	Report any additional statistics (e.g., assessments of heterogeneity across genetic variants, such as I^2, Q statistic or E-value)	7	F statistics for these IVs were calculated before MR analysis to determine if there was a weak IV bias. For all IVs, F>10, respectively, the effect of weak IV bias is little, so the selected SNPs can be further used in MR study.
13	**Sensitivity analyses and additional analyses**				
		a)	Report any sensitivity analyses to assess the robustness of the main results to violations of the assumptions	11	To further verify the reliability of the above results, we found no directional pleiotropy and heterogeneity was found by MR-Egger regression analysis (all P>0.05) (Figure 3), and funnel charts to visualize directional pleiotropy (Supplemental Figure 1).

		b) Report results from other sensitivity analyses or additional analyses	9, 10	Instrumental Variables for PUFAs and DR<br>Following a rigorous screening process based on the independence and exclusivity hypotheses, as well as the harmonization and removal of palindromic SNPs with intermediate allele frequencies, we meticulously selected 52 SNPs for the MR analysis to assess the associations between total PUFA and ADR. Similarly, for the evaluation of the relationship between ADR and total PUFA, 8 SNPs were selected as instrumental variables. The study also focused on investigating the causal effects of different types of PUFAs, including total PUFA, FAw3, FAw6, and FAw6/FAw3, on three DR phenotypes (BDR, SNPDR, and PDR) using MR analysis. In total, 34-57 SNPs were chosen for the MR analysis. The F-statistics of the instruments exceeded 10 for all selected SNPs, indicating satisfactory instrument strength. Detailed information regarding the instruments can be found in Supplemental Table 1.
		c) Report any assessment of direction of causal relationship (e.g., bidirectional MR)	10	Bidirectional causal effects between totally PUFAs and ADR<br>The results of MR showed that genetically predicted totally PUFA was associated with an decreased risk of ADR (IVW: OR, 0.90; 95% CI, 0.83-0.97; P=5.05×10-3). It can be seen from the scatter plot (Figure 2) that the causal effect among the three methods is consistent. However, no association of ADR with totally PUFAs was found using any method (all P>0.05). More MR analysis details are shown in Figure 3.
		d) When relevant, report and compare with estimates from non-MR analyses		
		e) Consider additional plots to visualize results (e.g., leave-one-out analyses)	11	Furthermore, no outliers were identified with MR-PRESSSO and the leave-one-out plot as well as funnel plots (Supplementary Figure 2).
	**DISCUSSION**			
14	**Key results**	Summarize key results with reference to study objectives	11	In this study, we utilized data from GWAS to perform bidirectional MR analyses based on the genetic associations of overall PUFAs and DR, and conducted two-sample MR study to examine the causal relationship between genetically determined PUFA features (overall PUFA, FAw3, FAw6,

				FAw6/FAw3) and three DR phenotypes (BDR, SNPDR, PDR).<br>Our main findings, along with sensitivity analyses, demonstrated a genetic predisposition to higher levels of overall PUFAs being associated with a reduced risk of DR. Reverse MR analysis did not provide any genetic evidence of ADR influencing PUFAs. The overall levels of PUFAs were associated with a decreased risk of BDR and PDR. Specifically, an elevation in FAw3 levels was found to be linked to a decreased risk of PDR. Similarly, an increase in FAw6 levels was associated with a reduced risk of both BDR and PDR.
15	**Limitations**	Discuss limitations of the study, taking into account the validity of the IV assumptions, other sources of potential bias, and imprecision. Discuss both direction and magnitude of any potential bias and any efforts to address them	15, 16	The study has some limitations. First, it should be noted that the selected GWAS data was all the results of a meta-analysis that had been adjusted for age and sex, and the data for ocular inflammation were all from the Finnish database. Although all the IVs we selected were strong, there is no denying that sample overlap can lead to bias. Secondly, our MR study showed that PUFAs predicted by genetics had a causal effect on DR, but the results of MR Analysis were only genetic evidence. Thirdly, we considered FAw3 or FAw6 components as a whole, so we need to further analyze the causality of individual FAw3 or FAw6 components separately. These limitations highlight the need for continued research to address the potential biases arising from sample overlap to further examine the causal relationship between PUFAs and DR through complementary experimental approaches, and to analyze the individual components of FAw3 and FAw6 in order to gain a more comprehensive understanding of their specific roles in the context of DR.
16	**Interpretation**			
	a)	Meaning: Give a cautious overall interpretation of results in the context of their limitations and in comparison with other studies	16, 17	Our findings present the initial genetic evidence supporting a causal association between PUFAs and the reduction of DR risk. Specifically, both FAw3 and FAw6 fatty acids demonstrate favorable effects on PDR, whereas FAw6 fatty acids play a mitigating role in BDR. However, neither of them exhibits a causal relationship with SNPDR. Nonetheless, the establishment of an effective therapeutic approach for the administration of

			FAw3 and FAw6 in the context of DR remains an unanswered and critical question. Consequently, our comprehensive MR analysis study strongly encourages further research to ascertain the precise roles and long-term effects of PUFAs, FAw3, and FAw6 in the treatment of DR.
b)	Mechanism: Discuss underlying biological mechanisms that could drive a potential causal relationship between the investigated exposure and the outcome, and whether the gene-environment equivalence assumption is reasonable. Use causal language carefully, clarifying that IV estimates may provide causal effects only under certain assumptions	13, 14	FAw3 predominantly encompass long-chain FAw3, such as eicosapentaenoic acid (EPA) and docosahexaenoic acid (DHA). Accumulating evidence suggests that supplementation with FAw3 benefits patients with DR. Nevertheless, no specific studies have investigated the relationship between FAw3 intake and different DR subtypes. Our MR study aims to address this knowledge gap. The findings indicated a potential mitigating effect of FAw3 on PDR, and FAw3 did not have a significant protective causal relationship between BDR and SNPDR, aligning with prior research that links increased FAw3 intake to a reduced risk of severe visual decline. Moreover, it has been demonstrated that FAw3 intake lowers the risk of pathological retinal neovascularization and severe visual impairment. Notably, lipotoxins, hemolysins, and protective proteins derived from FAw3 exhibiting anti-angiogenic properties and hold clinical potential in preventing diabetic macular edema and retinopathy.<br><br>The estimates provided in this study also take into account the long-term effects of FAw6, which remains an important question to be addressed. The evidence regarding the association between FAw6 levels and the risk of DR is limited and subjects to ongoing debate. Linoleic acid (LA), an FAw6 possesses anti-inflammatory properties and can modulate inflammation through various pathways, including immune cell function, and the inhibition of inflammatory mediators' production, among other actions. Arachidonic acid (AA), the other FAw6 plays a significant role in inflammatory responses and can be metabolized by enzymes such as cyclooxygenase and lipoxygenase to generate various inflammatory mediators, including prostaglandins and leukotrienes. In contrast to FAw3, FAw6 was considered pro-inflammatory properties, and maintaining a lower FAw6/FAw3 ratio is considered crucial for achieving favorable

			outcomes in ocular pathology. However, studies on healthy adults have demonstrated that increasing the intake of AA or LA does not necessarily result in elevated concentrations of many inflammatory markers. Epidemiological research has even suggested that AA and LA may be associated with reduced inflammation. Additionally, a study by Fu Z showed that a diet rich in FAw6 accelerated the maturation process of retinal neurons while inducing increased metabolism to maintain the energy balance of retinal neurons. The controversy surrounding FAw6 may arise from the differential roles of LA and AA within the eye. Research conducted by Kai Wang et al. found that LA is the major determinant explaining the inverse association between FAw6 and the risk of age-related macular degeneration, whereas increased levels of AA are causally associated with a higher risk of age-related macular degeneration.
c)	Clinical relevance: Discuss whether the results have clinical or public policy relevance, and to what extent they inform effect sizes of possible interventions	15	In our MR study, we suggest a consistent mitigating effect of FAw6, including LA and AA, on the risk of PDR. Interestingly, compared to FAw3, FAw6 also exhibit a mitigating effect on the risk of BDR, although neither of them has an impact on SNPDR. This suggests an overall beneficial role of FAw6 in DR, with specific mechanisms yet to be studied. The interaction between FAw3 and FAw6, as well as their lipid mediators, in the context of inflammation is complex and not yet fully understood. We did not find any significant influence of the FAw6 to FAw3 ratio on any subtype of DR.<br><br>It is worth noting that dietary sources of PUFAs are limited in Western diets, which highlights the importance of dietary interventions or nutritional supplementation, even in healthy individuals such as pregnant women, children, and the elderly. Therefore, implementing simple measures to enhance the production of endogenous anti-inflammatory molecules and adopting seemingly straightforward interventions throughout the entire course of DR in long-term diabetic patients could be beneficial. The establishment of a therapeutic approach for administering FAw3 in the treatment of DR remains an important unanswered question. Therefore, further research is encouraged to

				determine the specific role and long-term effects of FAw3 and FAw6 in the treatment of DR.
17	**Generalizability**	Discuss the generalizability of the study results (a) to other populations, (b) across other exposure periods/timings, and (c) across other levels of exposure	16, 17	In conclusion, our findings present the initial genetic evidence supporting a causal association between PUFAs and the reduction of DR risk. Specifically, both FAw3 and FAw6 fatty acids demonstrate favorable effects on PDR, whereas FAw6 fatty acids play a mitigating role in BDR. However, neither of them exhibits a causal relationship with SNPDR. Nonetheless, the establishment of an effective therapeutic approach for the administration of FAw3 and FAw6 in the context of DR remains an unanswered and critical question. Consequently, our comprehensive MR analysis study strongly encourages further research to ascertain the precise roles and long-term effects of PUFAs, FAw3, and FAw6 in the treatment of DR.
	**OTHER INFORMATION**			
18	**Funding**	Describe sources of funding and the role of funders in the present study and, if applicable, sources of funding for the databases and original study or studies on which the present study is based	17	Funded by Tianjin Key Medical Discipline (Specialty) Construction Project (TJYXZDXK-037A)
19	**Data and data sharing**	Provide the data used to perform all analyses or report where and how the data can be accessed, and reference these sources in the article. Provide the statistical code needed to reproduce the results in the article, or report whether the code is publicly accessible and if so, where	17	The datasets analysed during the current study are available in the https://gwas.mrcieu.ac.uk and https://msk.hugeamp.org.
20	**Conflicts of Interest**	All authors should declare all potential conflicts of interest	17, 18	All authors declare that we have no financial and personal relationships with other people or organizations that can inappropriately influence our work and that there is no professional or other personal interest of any nature or kind in any product, service and/or company that could be construed as influencing the position presented in, or the review of, the manuscript entitled "Causal Associations Between Polyunsaturated Fatty Acids and Diabetic Retinopathy: A Two-Sample Mendelian Randomization Study".

This checklist is copyrighted by the Equator Network under the Creative Commons Attribution 3.0 Unported (CC BY 3.0) license.

1. Skrivankova VW, Richmond RC, Woolf BAR, Yarmolinsky J, Davies NM, Swanson SA, et al. Strengthening the Reporting of Observational Studies in Epidemiology

using Mendelian Randomization (STROBE-MR) Statement. JAMA. 2021;under review.

2. Skrivankova VW, Richmond RC, Woolf BAR, Davies NM, Swanson SA, VanderWeele TJ, et al. Strengthening the Reporting of Observational Studies in Epidemiology using Mendelian Randomisation (STROBE-MR): Explanation and Elaboration. BMJ. 2021;375:n2233.
