# Supplementary material for: Mendelian Randomization Analysis Reveals Causal Effects of Polyunsaturated Fatty Acids on Subtypes of Diabetic Retinopathy Risk
Source: Nutrients. 2023 Sep 29;15(19):4208. doi: 10.3390/nu15194208 (PMC10574403; doi:10.3390/nu15194208)
Supplement: Supplementary file 1 [file nutrients-15-04208-s001.zip › Supplemental Figure.pdf]

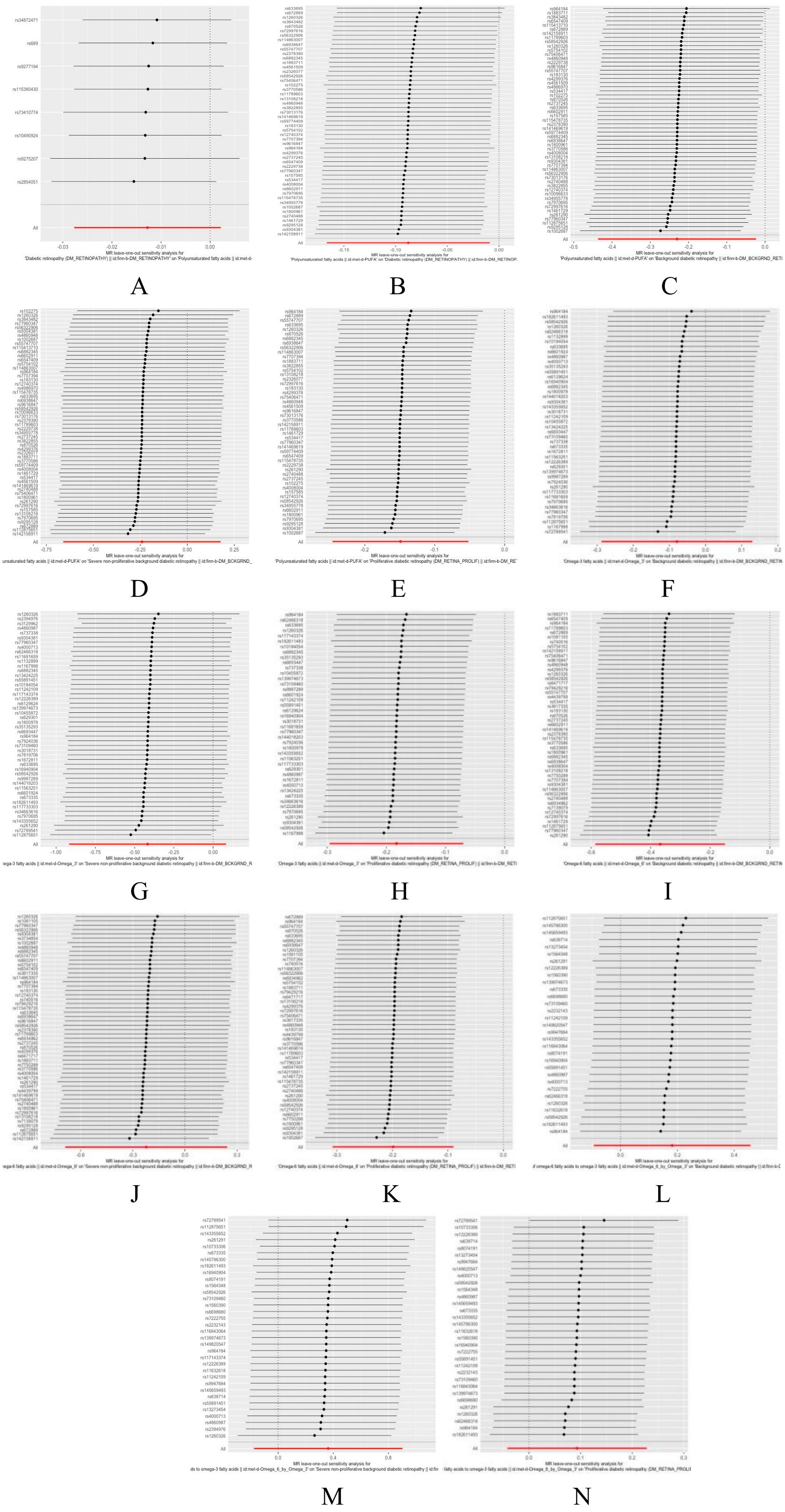

Supplementary Figure S1. Leave one out sensitivity tests of MR analyses of PUFAs and DR. Calculate the MR results of the remaining IVs after removing the IVs one by one. A: ADR on totally PUFAs; B: totally PUFAs on ADR; C: totally PUFAs on BDR; D: totally PUFAs on SNPDR; E: totally

PUFAs on PDR; F: FAw3 on BDR; G: FAw3 on SNPDR; H: FAw3 on PDR; I: FAw6 on BDR; J: FAw6 on SNPDR; K: FAw6 on PDR; L: FAw6/FAw3 on BDR; M: FAw6/FAw3 on SNPDR; N: FAw6/FAw3 on PDR.

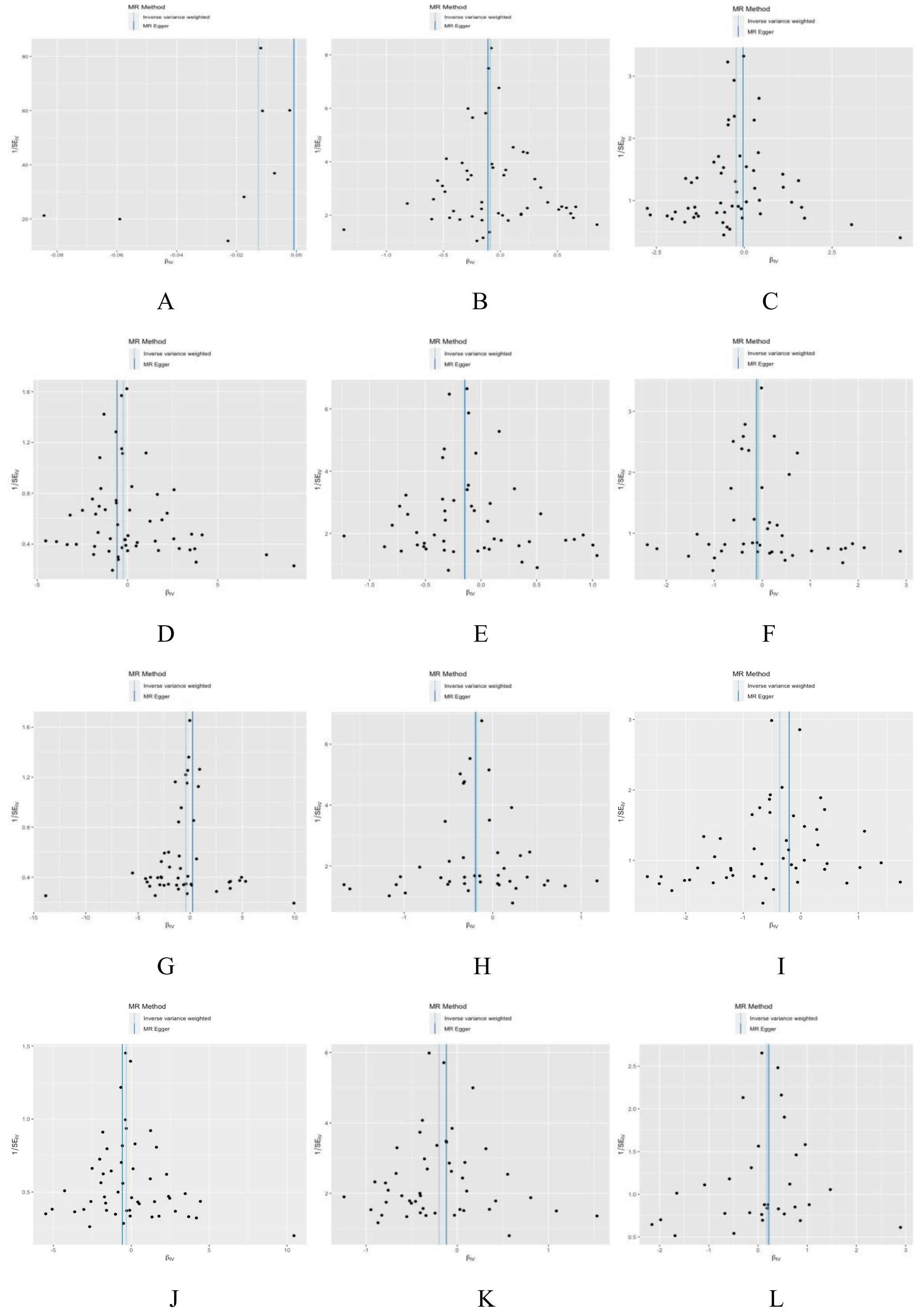

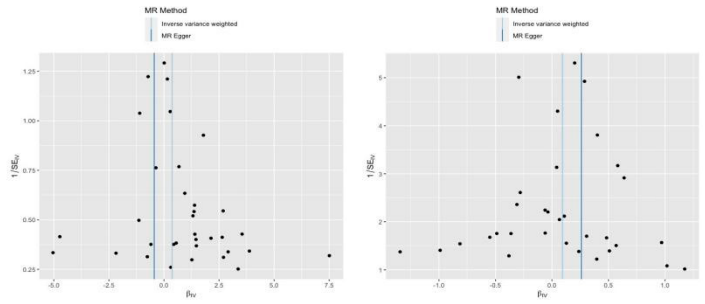

M N

Supplementary Figure S2. Funnel plot of MR analyses of PUFAs and DR. Calculate the MR results of the remaining IVs after removing the IVs one by one. A: ADR on totally PUFAs; B: totally PUFAs on ADR; C: totally PUFAs on BDR; D: totally PUFAs on SNPDR; E: totally PUFAs on PDR; F: FAw3 on BDR; G: FAw3 on SNPDR; H: FAw3 on PDR; I: FAw6 on BDR; J: FAw6 on SNPDR; K: FAw6 on PDR; L: FAw6/FAw3 on BDR; M: FAw6/FAw3 on SNPDR; N: FAw6/FAw3 on PDR.
